# Fine‐Scale Genetic Structure of Small Fish Populations in Islands: The Case of Brook Charr *Salvelinus fontinalis* (Mitchill, 1814) in Saint‐Pierre and Miquelon (France)

**DOI:** 10.1111/eva.70041

**Published:** 2025-01-16

**Authors:** Julie Viana, Guillaume Evanno, Céline Audet, Fabrice Teletchea

**Affiliations:** ^1^ Laboratoire Animal et Agroécosystèmes—L2A Université de Lorraine Vandœuvre‐Lès‐Nancy France; ^2^ Institut des sciences de la mer de Rimouski Université du Québec à Rimouski Rimouski Québec Canada; ^3^ DECOD (Ecosystem Dynamics and Sustainability) INRAE, Institut Agro, IFREMER Rennes France; ^4^ UMR CNRS 7360, LIEC, Laboratoire Interdisciplinaire Des Environnements Continentaux Université de Lorraine Nancy France

**Keywords:** dispersal, microsatellites markers, salmonids, *Salvelinus fontinalis*, small populations, stocking

## Abstract

Island ecosystems, particularly vulnerable to environmental challenges, host many endangered native species. Diadromous fish, in particular, are threatened throughout their marine and freshwater habitats. The conservation of these species requires an in‐depth understanding of their genetic diversity and structure, to better understand their adaptive potential. We investigated fine‐scale population diversity and structure in native brook charr (*Salvelinus fontinalis*) by genotyping 10 microsatellite *loci* in 244 individuals at three spatial scales in Saint‐Pierre and Miquelon, France. We found limited genetic variability across the archipelago, with particularly low genetic diversity in one island, Langlade. A significant difference in allelic richness was also detected among the three islands, indicating a difference in genetic composition across the archipelago, probably induced by historical stocking actions on both Saint‐Pierre and Miquelon. Finally, a strong genetic structure was detected across the archipelago among hydrosystems (overall *F*_ST_ = 0.19) and even within several of them. The presence of predominant interisland gene flow combined with complete genetic isolation from certain hydrosystems suggests that this contemporary genetic structure is the result of both natural demographic processes during the species postglacial colonization and recent restocking actions. The complex genetic structure of such isolated brook charr subpopulations highlights the importance of considering fine‐scale genetic structure in conservation management.

## 1 Introduction

The persistence of populations in changing environments is directly correlated with genetic diversity (Dobzhansky 1955; Kardos et al. 2021). This variability constitutes an essential reservoir for evolutionary processes, including natural selection (Saccheri et al. 1998; Labonne et al. 2016). On islands, populations generally originate from a small number of founder individuals, which, by adapting to unique environmental conditions, may evolve toward endemic island forms (White and Searle 2007). The low number of founder individuals, coupled with small population size, makes such populations more vulnerable and more sensitive to demographic and environmental stochasticity compared to continental conspecific populations (Gilpin and Soulé 1986; White and Searle 2007; Bernatchez 2016). Accordingly, their genetic diversity is strongly influenced by both genetic drift and migration limitations (Kapralova et al. 2011). The existence of sufficient gene flow among such small populations may, however, enable them to persist. Assessing connectivity patterns should therefore be a conservation priority, particularly for small island populations (Tian et al. 2022; Ash et al. 2023).

Brook charr, *Salvelinus fontinalis* (Mitchill 1814), is an emblematic salmonid of North America, present in almost every type of cold‐water habitats, from streams and rivers to lakes and nearshore marine environments (Behnke 1972). It is one of the most popular fish for recreational fishing (Quigley 2015) and a good indicator of habitat quality (Dutil and Power 1980). In recent decades, a general decline in wild populations has been documented (Hudy et al. 2008; Stranko et al. 2008; EPA 2015), chiefly due to habitat degradation (e.g., deforestation, fragmentation), overfishing, introduction of non‐native competitors or parasitism (Ryther 1997; Miller et al. 2016; Caputo et al. 2017). The decline is set to worsen in coming years due to climate change (Jeppesen et al. 2015; Al‐Ghussain 2019; Alfonso, Gesto, and Sadoul 2021), which may overwhelm the adaptive response capacity of these organisms (Bassar et al. 2016; Lynch et al. 2016; Poesch et al. 2016).

The native range of brook charr extends from the southern Appalachians to the northern Canadian Maritimes and west to the Hudson Bay watershed (MacCrimmon and Campbell 1969; Dutil and Power 1980). This distribution and the current genetic structure result from the retreat of ice caps at the end of the last major glacial episode in North America (McPhail and Lindsey 1986; Dyke and Prest 1987; Pielou 1991; Hewitt 1996). The anadromy potential of this species is thought to have played a major role in the recolonization of North American rivers. This ancestral trait is expressed as soon as the habitat is connected to the ocean (Curry et al. 2010). A freshwater resident form may appear and it is now possible to observe allopatric resident populations as well as sympatric resident and anadromous populations (Perry et al. 2005; Thériault, Bernatchez, and Dodson 2007; Wilson et al. 2008). The brook charr ranks among the most highly genetically structured animal species (Gyllensten 1985; Ward, Woodwark, and Skibinski 1994; Castric, Bonney, and Bernatchez 2001), with most of its genetic variance partitioned among major drainage (Ferguson, Danzmann, and Hutchings 1991; Perkins, Krueger, and May 1993; Danzmann and Ihssen 1995; Angers and Bernatchez 1998).

Saint‐Pierre and Miquelon is a French archipelago located 25 km southwest of the Canadian province of Newfoundland (Teletchea 2022) and constitutes the easternmost limit of the brook charr native range (Viana et al. 2022). Postglacial recolonization of the region by this species is thought to have taken place from a glacial refuge along the southeast coast of Newfoundland, known as the Acadian Refuge (Schmidt 1986). Since 2000s, a strong decline of the archipelago populations has been observed (Champigneulle, Moutonet, and Gerdeaux 2000; Gerdeaux 2000; Cloutier, Lemay, and Gerdeaux 2003; Preynat 2013). Brook charr from Saint‐Pierre and Miquelon could either be resident or anadromous, but connectivity among hydrosystems has never been investigated (Viana et al. 2022). Up till now, there has been no scientifically based management of these populations. Brook charr is the only species with an angling interest among the seven diadromous fish inhabiting this archipelago (Denys et al. 2022). Quotas are set for recreational fisheries, but they do not seem to be based on any solid scientific foundation even though some rules have been established over time (Briand et al. 2021). In addition, there were some stocking activities without taking into account neither the source population nor the localization of stockings (Briand et al. 2021; Viana et al. 2022). For these reasons, the genetic integrity of the different populations is unknown.

In this study, we aimed at evaluating for the first time ever, the genetic diversity and population genetic structure of the brook charr in Saint‐Pierre and Miquelon using microsatellite markers at three spatial scales: islands, rivers, and positions within these rivers. This data should provide key information to support future management and conservation actions of these small populations.

## 2 Methods

### 2.1 Study Sites and Sample Collection

**FIGURE 1:**
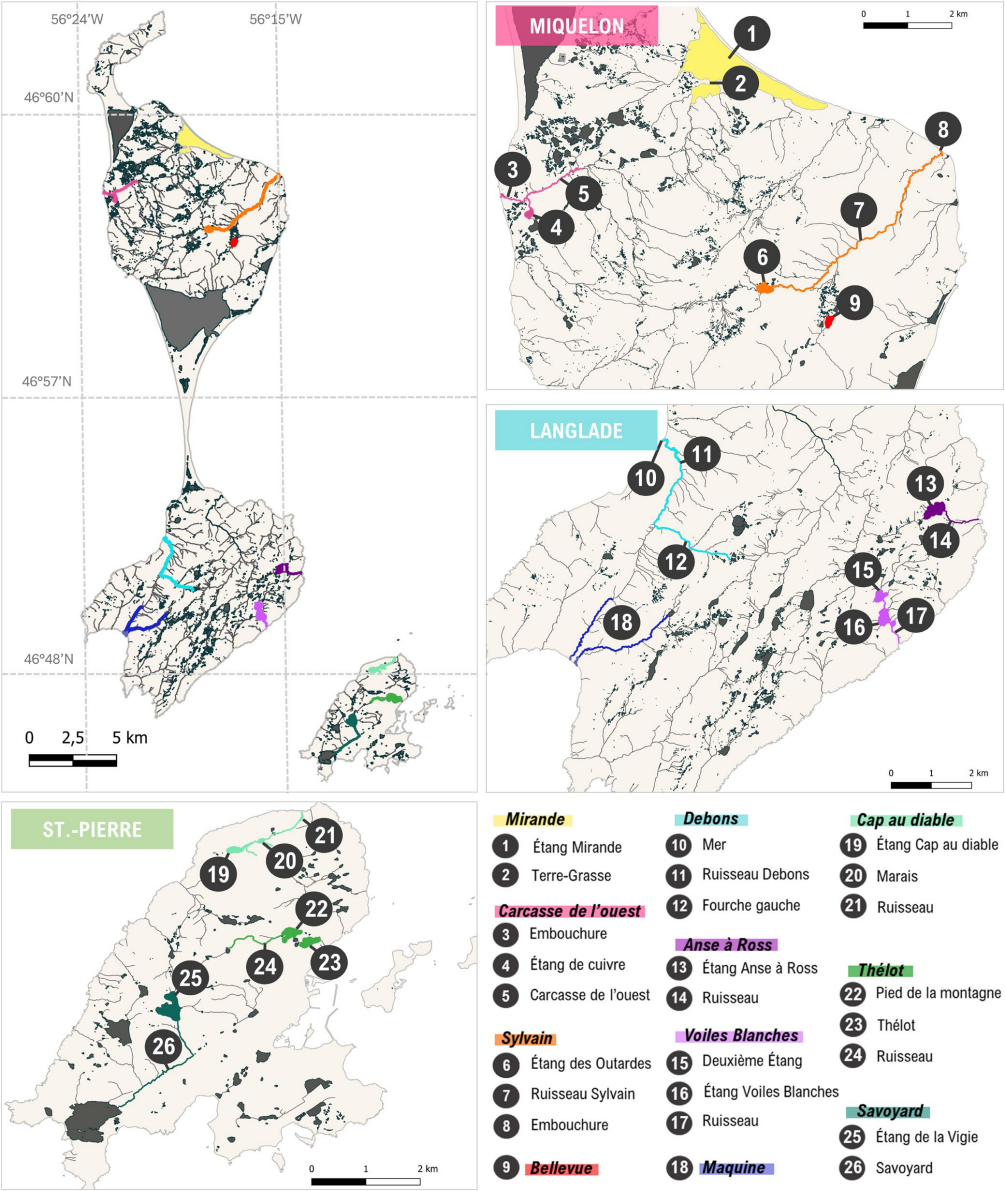
Saint‐Pierre and Miquelon, France. The 11 hydrosystems studied are shown in color in the insets of their respective islands. The sites sampled within each hydrosystem are numbered from 1 to 26. The names of each hydrosystem and site are detailed in the legend at the bottom right.

We selected 11 hydrosystems belonging to distinct watershed distributed across the three main islands of the archipelago (Figure 1). In Miquelon, the first site was *Mirande* pond, previously open to the ocean, but closed by a road construction in the 1960s (Briand et al. 2021). The three other hydrosystems targeted on this island (*Carcasse de l'ouest*, *Sylvain* and *Bellevue*) could be considered relatively pristine. Langlade is the wildest island (human population only during spring and summer) hosting fish that seem to have developed unique characteristics: *Anse à Ross*, small individuals; *Voiles Blanches*, colorful individuals; *Debons*, the largest yearly captures; and *Maquine*, a very isolated site (Briand et al. 2021). On Saint‐Pierre, the smallest and most impacted island by human activities, several hydrosystems have been strongly modified (e.g., dams or destruction of the watershed), which prevents the upstream migration of anadromous brook charr. The three sampled hydrosystems were *Savoyard* (dam in the upstream part of the hydrosystem), *Thélot* (two former hydroelectric dams), and *Cap au diable* (isolated sites due to an insurmountable waterfall close to the coastal area; Figure 1).

We aimed to capture a total of 30 individuals per hydrosystems (10 in the upstream part, 10 in the median part, and 10 in the estuarine part) by electrofishing or traditional angling techniques (i.e., cork, spinner, or whip fishing). Fish captures were performed in each targeted sampled area so that we cover the entire area as much as possible.

Captures were made according to CCAC (UQAR CPA‐89‐22‐246) and the prefectural permit (no. 349). Following the capture, fish were anesthetized with eugenol 100% (Lotus Aroma, Sainte‐Julie, QC, Canada) and killed by sectioning the spinal cord. Tissues were sampled for further molecular analyses. The adipose fin was cut and preserved in 95% ethanol.

### 2.2 DNA Extraction and Genotyping

Genomic DNA was extracted from 2 mm of fin tissue using the QIAGEN “DNeasy Blood and tissue” kit (Qiagen, Hilden, Germany). The extractions were carried out according to the manufacturer recommendations by adjusting the elution volume to 100 μL. After preliminary tests on amplification, 12 microsatellite loci were kept (*Sfo177Lav*, *Sfo308Lav*; Perry et al. 2005 and *SfoB52*, *SfoC38*, *SfoC86*, *SfoC88*, *SfoC113*, *SfoC115*, *SfoC129*, *SfoD75*, *SfoD91*, *SfoD100*; King et al. 2012), and amplified using fluorescently labelled primers (6‐FAM, ATTO 532, ATTO 550, ATTO 565, Applied Biosystems). Microsatellite primer sequences are provided in Table S1. Polymerase chain reactions (PCR) were performed using the QIAGEN “Type‐it Microsatellite PCR” kit (Qiagen, Hilden, Germany). Amplifications were carried out as follows: 5 min at 95°C, followed by 40 cycles of 30 s at 95°C, 1 min 30 s at optimal annealing temperature, 30 s at 72°C and final extension for 30 min at 60°C. Loci were combined in four multiplexes according to their size range and primer annealing temperature (*T*_a_) to perform PCR (Table S1). PCR products were run through 2.0% agarose gel to test the success of amplification and sent to GenoScreen (Lille, France) for genotyping. Allele sizes were determined by Geneious Prime 2023.1.2 software (https://www.geneious.com). Missing data represented less than 1% of the total data set so all individuals were considered for analyses.

### 2.3 Genetic Diversity

The presence of null alleles and large allelic dropouts were evaluated using MICRO‐CHECKER v.2.2.3 (Oosterhout et al. 2004). A loci‐pair approach implemented in Genepop v4.0 (Rousset 2008) was used to determine the loci involved in potential linkage disequilibrium (*LD*).

Genetic diversity, including estimates of expected (*H*_E_) and observed (*H*_O_) heterozygosity, was calculated for each hydrosystem using GENETIX v.4.05 software (Belkir et al. 1996). Measures of the allelic richness (Ar), inbreeding coefficient (*F*_IS_; Weir and Cockerham 1984) and tests for Hardy–Weinberg disequilibrium (3000 permutations) were calculated in each hydrosystem using FSTAT v2.9.4 (Goudet 2003). We also compared the allelic richness among the islands (Ar), using FSTAT with 5000 permutations. When a high number of full‐sibling relationships were detected in a given hydrosystem (see below), diversity indices were recalculated by excluding related individuals to determine their influence on these estimates.

### 2.4 Kinship

To compare the level of relatedness (*R*) among islands, we used an estimator strictly equivalent to Queller and Goodnight (1989) implemented in FSTAT (Goudet 2003). The significance of the comparison was assessed based on 5000 permutations. At a finer scale, we computed kinship relationships within each hydrosystem using COLONY v2.0.6.6 (Jones and Wang 2010). This program used the full maximum likelihood approach to assign individuals to full‐siblings or half‐siblings. For this analysis, a conservative genotyping error of 5% was used. The multinominal a priori was implemented to reduce the risk of nonrelated or weakly related individuals being falsely detected, which is recommended when both sexes are polygamous as in brook charr (Wang 2004).

### 2.5 Population Genetic Structure

A Bayesian clustering approach was performed using STRUCTURE v.2.3.4 software (Pritchard, Stephens, and Donnelly 2000) to identify the number of genetically distinct clusters (*K*) and for inferring admixture. For each value of *K*, 10 iterations were run to assess the convergence of the likelihood with a burn‐in period of 100,000 followed by 100,000 iterations for values of *K* = 1 through 11. Each simulation was performed with an ancestry model incorporating admixture, a model of correlated allele frequencies, and without prior population information. To determine the highest hierarchical level of genetic structure in the data set, the average likelihood value for each *K* and the Δ*K* curve were analyzed using Evanno's method (Evanno, Regnaut, and Goudet 2005) implemented in STRUCTURE HARVESTER (Earl and vonHoldt 2012). In addition, a Discriminant Principal Component Analysis (DAPC) was performed using the R *adegenet* package (Jombart 2008) to assess the optimal number of genetic clusters, based on the α‐score and considering the island where each individual was sampled. This method differs from STRUCTURE analyses in that it does not rely on criteria like Hardy–Weinberg equilibrium or *LD* to delineate genetic clusters. It can therefore be more effective in identifying genetic clines and hierarchical structures (Jombart, Devillard, and Balloux 2010).

To analyze the hierarchical structure we performed an Analysis of Molecular Variance (AMOVA) using ARLEQUIN 3.5.2.2 (Excoffier and Lischer 2010). This analysis allowed partitioning the genetic variability at different spatial scales: among islands, between hydrosystems within islands and within hydrosystems. In addition, an overall estimate of *F*_ST_ (*θ*) among the 11 hydrosystems was calculated using FSTAT v.2.9.4 (Weir and Cockerham 1984). Pairwise *F*_ST_ were computed with GENETIX v.4.05 software (Belkir et al. 1996) using the W&C estimator for interisland and interhydrosystem analyses. The Robertson and Hill estimator corrected by Raufaste and Bonhomme (RH') was used for within‐hydrosystem comparisons because of the small size of populations at this scale. Indeed, the RH' estimator is more accurate at detecting genetic differentiation in very small populations, for which genetic drift is generally stronger and genetic differentiation weaker (Raufaste and Bonhomme 2000). All *p‐values* were assessed based on 3000 permutations.

An isolation by distance (IBD) analysis was carried out using the “*vegan*” package of R software (Oksanen et al. 2024), using pairwise *F*_ST_ values among hydrosystems and the coastal distance between the mouths of the rivers studied. The IBD model was analyzed using linear regression of *F*_ST_/1‐*F*_ST_ as a function of log (distance) following the method of Rousset (1997).

## 3 Results

Overall, 244 brook charr were collected in Saint‐Pierre (*n* = 75), Miquelon (*n* = 79), and Langlade (*n* = 90) from May to August 2022 (Table 1).

**TABLE 1:** Estimated genetic diversity for the brook charr populations of Saint‐Pierre and Miquelon, including sample size (*N*), expected (*H*_E_) and observed (*H*_O_) heterozygosity, allelic richness (Ar) and Wright's inbreeding coefficient (*F*_IS_). Significant *F*_IS_ values are indicated by an asterisk (*p < 0.05; **p < 0.005). Genetic diversity indices for *Bellevue* (Miquelon), *Voiles Blanches* and *Anse à Ross* (Langlade) shown in bold and in parentheses correspond to those calculated without related individuals (see 2. Methods section).

Island (*N*)	Hydrosystem (*N*)	Site (*N*)	*H*_E_	*H*_*O*_	Ar	*F*_IS_
Saint‐Pierre (75)	Cap au diable (26)	Étang Cap au diable (10)	0.503	0.504	4.16	0.02
		Marais (10)				
		Ruisseau (6)				
	Thélot (29)	Pied de la montagne (9)	0.544	0.545	4.12	0.02
		Thélot (10)				
		Ruisseau (10)				
	Savoyard (20)	Étang de la Vigie (10)	0.559	0.550	4.89	0.04
		Savoyard (10)				
Miquelon (79)	Mirande (20)	Étang Mirande (10)	0.456	0.412	3.49	0.12*
		Terre‐Grasse (10)				
	Carcasse de l'ouest (19)	Embouchure (6)	0.472	0.425	3.74	0.13**
		Étang de cuivre (3)				
		Carcasse de l'ouest (10)				
	Sylvain (30)	Étang des Outardes (10)	0.508	0.494	3.68	0.05
		Ruisseau Sylvain (10)				
		Embouchure (10)				
	Bellevue (10–**8**)		0.321 **(0.343)**	0.330 **(0.338)**	2.40 **(2.40)**	0.02 **(0.08)**
Langlade (90)	Debons (28)	Mer (9)	0.400	0.461	3.72	0.15**
		Ruisseau Debons (10)				
		Fourche gauche (10)				
	Anse à Ross (20–**14**)	Étang Anse à Ross (10)	0.239 **(0.265)**	0.228 **(0.248)**	1.80 **(1.77)**	0.07 **(0.10)**
		Ruisseau (10)				
	Voiles Blanches (26–**18**)	Deuxième Étang (10)	0.191 (**0.223**)	0.177 (**0.217**)	1.86 (**1.89**)	0.09 (**0.06**)
		Voiles Blanches (10)				
		Ruisseau (7)				
	Maquine (16)		0.414	0.412	2.95	0.04

### 3.1 Genetic Variability

Evidence of null alleles or large allele dropouts was detected for two loci (*SfoC88* and *Sfo177Lav*) with MICRO‐CHECKER analyses hence those loci were discarded for further analyses. Linkage disequilibria were also detected in four hydrosystems (Table S2). As none of these loci showed a recurrence of linkage disequilibrium in all hydrosystems, all 10 loci were kept for further analyses.

A total of 92 alleles over the 10 microsatellite loci was found in the full data set. The most diverse locus (*Sfo308Lav*) had 18 alleles and the least diverse (*SfoC38*) had 4. Expected and observed heterozygosity (*H*_E_ and *H*_O_) were moderate (mean *H*_E_ = 0.424; mean *H*_O_ = 0.407), from *H*_E_ = 0.191 and *H*_O_ = 0.177 in *Voiles Blanches* to *H*_E_ = 0.559 and *H*_O_ = 0.550 in *Savoyard* (Table 1). Similarly, the highest allelic richness was found in the *Savoyard* hydrosystem in Saint‐Pierre (Ar = 4.887), and the lowest in the *Anse à Ross* hydrosystem in Langlade (Ar = 1.796; Table 1). *F*_*IS*_ estimates were positive in all hydrosystems (mean = 0.07, from 0.02 to 0.15) and significant in three of them (Table 1). Overall, comparison of allelic richness among islands revealed populations sampled in Saint‐Pierre displayed significantly higher levels of allelic richness, followed by those in Miquelon and then Langlade (Ar_St.‐Pierre_ = 4.39; Ar_Miquelon_ = 3.33; Ar_Langlade_ = 2.58; *p* < 0.05). Three hydrosystems showed a significant number of full sibs (*see* 3.2 kinship results below). Computing the diversity indices by excluding those individuals revealed minor changes in these estimates (Table 1); hence, all individuals were retained for further analyses.

### 3.2 Kinship

The relatedness differed among islands as brook charr from Langlade were significantly more related than in Miquelon and Saint‐Pierre (*R*_St.‐Pierre_ = 0.05; *R*_Miquelon_ = 0.19; *R*_Langlade_ = 0.51; *p* < 0.05). Analyses with COLONY revealed few full sibling relationships (44 different individuals; Table 2) except in three hydrosystems: *Bellevue* (Miquelon), *Voiles Blanches* and *Anse à Ross* (Langlade) where 6, 31 and 21 such relationships were detected respectively (Table 2).

**TABLE 2:** Results of the COLONY analysis of sibling relationships of Saint‐Pierre and Miquelon brook charr according to their hydrosystems. (*N*) Indicates the number of different individuals involved in the relationships; values indicate the number of full sibling relationships detected within and among sampling locations. Half sibling relationships are indicated by an asterisk.

Locations (*N*)	CCO (2)	BLL (4)	DB (4)	AR (12)	VB (20)	MQ (4)	SVY (1)	CAP (1)
Carcasse de l'Ouest (CCO, Miq.)	1							
Bellevue (BLL, Miq.)	—	6						
Debons (DB, Lang.)	—	—	2					
Anse à Ross (AR, Lang.)	—	—	—	21				
Voiles Blanches (VB, Lang.)	—	—	—	—	31			
Maquine (MQ, Lang.)	—	—	—	—	—	2		
Savoyard (SVY, Lang.)	—	—	—	—	—	—	—	
Cap au diable (CAP, St.‐P.)	—	—	—	—	—	—	1*	—

*Note:* The sum of the sibling relationships can be larger than the sample sizes as individuals can have sibling relationships with more than one other individual.

Abbreviations: Lang.: Langlade, Miq.: Miquelon, St.‐P.: Saint‐Pierre.

### 3.3 Population Structure Among Hydrosystems and Between Islands

**FIGURE 2:**
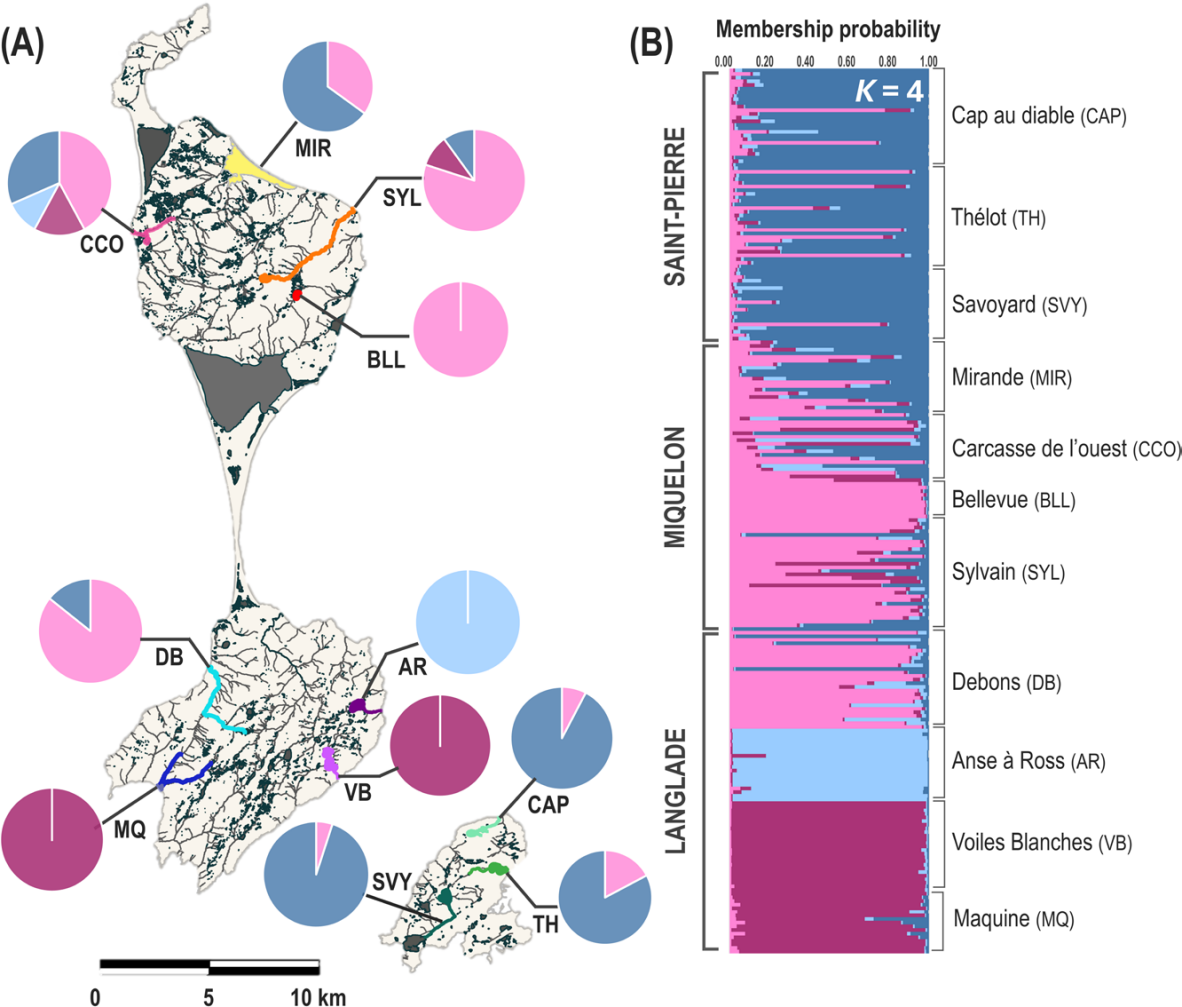
(A) Mapping charts of estimated group membership coefficients for all hydrosystems studied. Hydrosystem acronyms are shown next to the corresponding bar plot. Details of the acronyms are available on the STRUCTURE bar plots. (B) The colors correspond to the STRUCTURE bar plots obtained for all individuals as a function of their sampling hydrosystem for *K* = 4.

The highest level of genetic structure detected by STRUCTURE was *K* = 4 with two clusters corresponding roughly to Saint‐Pierre and Miquelon islands, whereas the Langlade Island displayed two distinct clusters and some individuals assigned to the Miquelon cluster (Figure 2B and Figure S1).

**FIGURE 3:**
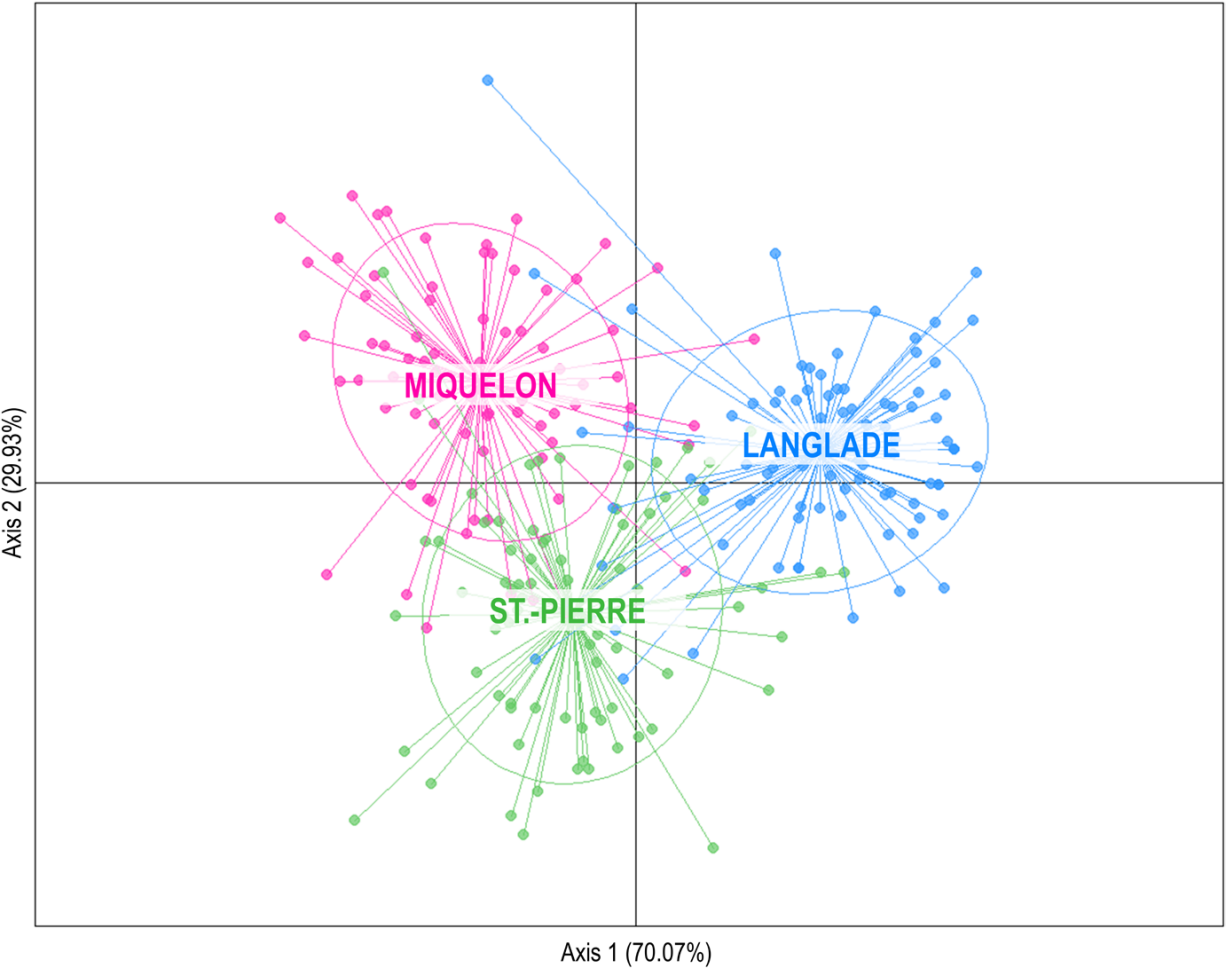
Ordination plots of Discriminant Principal Component Analysis (DAPC) comparing all individuals included in the study according to their island retaining 20 principal components. Inferred genetic clusters corresponding to each sampled island are shown using colors and inertia ellipses. Each dot represents an individual fish. The percentage of variance explained is indicated below each axis in parentheses.

The AMOVA analysis revealed that only 2.56% of the total variance was attributed to (nonsignificant) differences among islands (Table 3) even if three genetic clusters were clearly highlighted by the DAPC analysis (Figure 3). The proportion of the variance explained by differences among hydrosystems within the islands was high (17.66%; Table 3) but most of the total genetic variance was found within hydrosystems (79.78%; Table 3).

**TABLE 3:** AMOVA analysis with partitioning of genetic variance among islands, among hydrosystems within islands and within hydrosystems.

Source of variation	Degree of freedom	Sum of squares	Components of variance	Percentage of variation
Among islands	2	69.41	0.06	2.56^NS^
Among hydrosystems within islands	8	180.00	0.47	17.66^***^
Within hydrosystems	477	1041.5	2.18	79.78^***^

Abbreviation: NS, not significant.

*** *p* < 0.0001.

**FIGURE 4:**
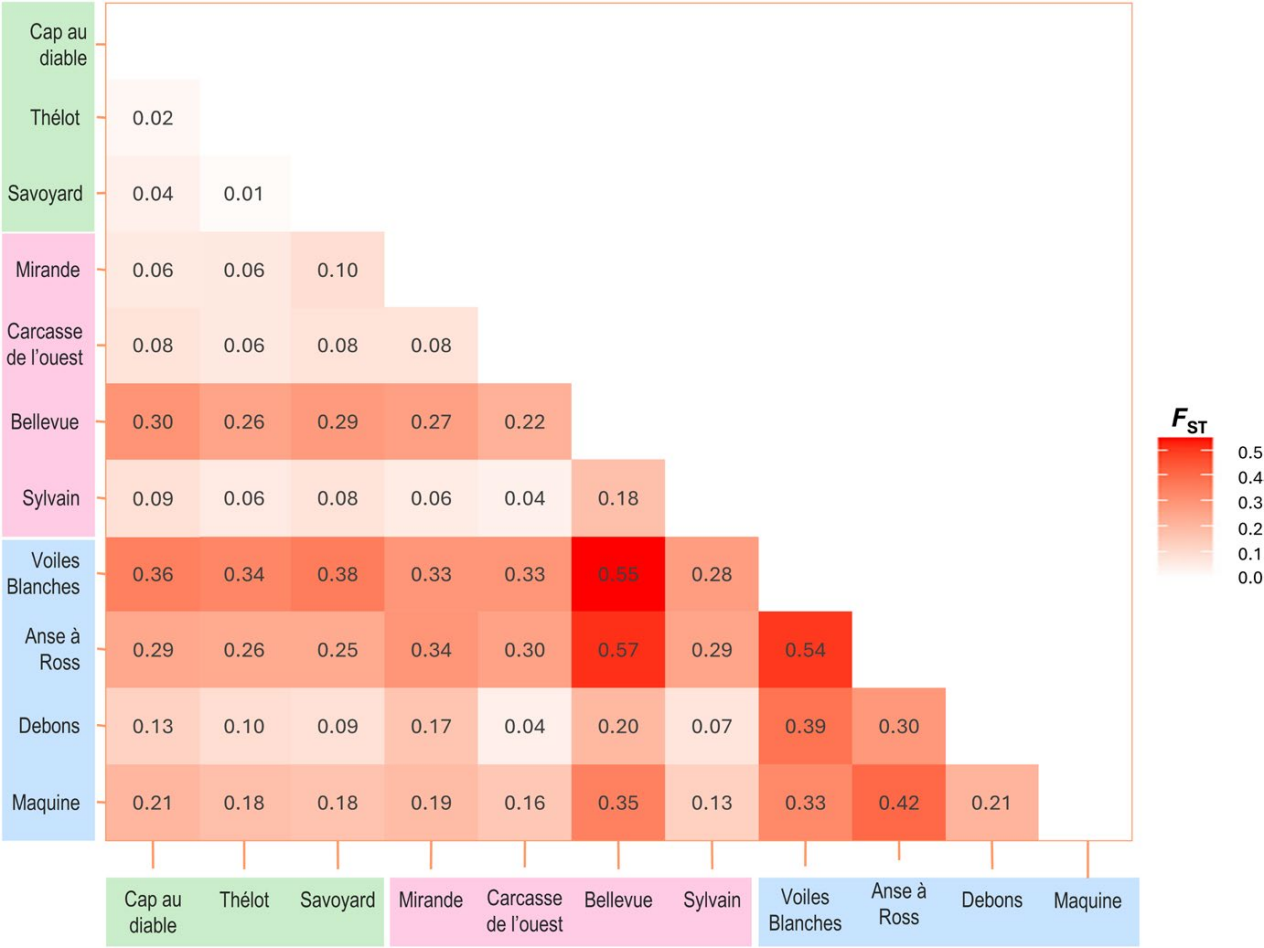
Pairwise *F*_ST_ values among all hydrosystems studied, calculated using Weir and Cockerham's unbiased *F*_ST_ estimator. All values presented here are significant (*p* < 0.05).

Accordingly, the level of genetic differentiation between brook charr from the three islands was moderate with pairwise *F*_ST_ of 0.06 between Saint‐Pierre and Miquelon, and between Miquelon and Langlade, to above 0.09 between Saint‐Pierre and Langlade (*p* < 0.0001, Figure 4).

The overall *F*_ST_ (θ) among hydrosystems was high: 0.19 (95% CI: 0.15–0.24; pairwise *F*_ST_: 0.01–0.57; Figure 4). Four hydrosystems from Langlade were highly differentiated from the others: *Bellevue* Pond, *Voiles Blanches*, *Anse à Ross*, and *Maquine* (Figure 4). Finally, no pattern of isolation by distance was detected among hydrosystems (*p* = 0.786; Figure S2).

### 3.4 Population Structure Within Hydrosystems

A significant genetic structure was detected within three hydrosystems: *Sylvain* (Miquelon), *Cap au diable* and *Thélot* (Saint‐Pierre). In *Sylvain*, genetic differentiation was observed between *Étang des Outardes*, located upstream of the hydrosystem, and the river (median portion; *F*_ST_ = 0.08, *p* < 0.05), and between *Étang des Outardes* and the estuary sampling site, with an *F*_ST_ of 0.06 (*p* < 0.05). In *Cap au diable*, the marsh (middle section of the hydrosystem) and the river (downstream part) populations were differentiated, with an *F*_ST_ of 0.08 (*p* < 0.05). In *Thélot*, an *F*_ST_ of 0.06 (*p* < 0.05) was found between populations from the middle part called *Étang du Pied de la Montagne* and the river (upstream of the hydrosystem).

## 4 Discussion

The aim of this study was to assess the diversity and genetic structure of Saint‐Pierre and Miquelon brook charr at three spatial scales to determine contemporary genetic connectivity and discuss the possible vulnerability of these small, isolated populations to climate change and other anthropogenic disturbances. Overall, we found (1) a limited genetic variability with significant differences among the three islands: the highest allelic richness was detected in the smallest island of Saint‐Pierre and the lowest diversity in Langlade, and (2) a high genetic structure across all spatial scales: among islands, hydrosystems and even within hydrosystems despite the small size of Saint‐Pierre and Miquelon archipelago.

### 4.1 Different Levels of Genetic Diversity Among Islands

The average level of expected heterozygosity in these brook charr populations is in the lower portion of the spectrum of variability observed throughout the northeastern range (Angers and Bernatchez 1998, *H*_E_ = 0.762; Adams and Hutchings 2003, *H*_E_ = 0.690; Rogers and Curry 2004, *H*_E_ = 0.775; Poissant, Knight, and Ferguson 2005, *H*_E_ = 0.380; Pilgrim et al. 2012, *H*_E_ = 0.620); yet such comparisons should be taken with caution, because we analyzed a different panel of microsatellite markers than the ones used in the former studies. Heterozygosity and allelic richness levels were particularly low in Langlade as well as in one hydrosystem of Miquelon (*Bellevue*). Accordingly, the highest level of relatedness was found in Langlade, especially in *Anse à Ross* and *Voiles Blanches* hydrosystems. These patterns of low diversity and high relatedness suggest low population size. These populations are also highly differentiated from other hydrosystems, indicating a low connectivity probably due to environmental factors.

The hydrosystems of *Mirande*, *Carcasse de l'ouest* (Miquelon), and *Debons* (Langlade), also show some degree of inbreeding, even though several of them have a connection to the ocean that could, in principle, allow potential exchanges with other populations. These significant *F*_IS_ values may also be explained by a Wahlund effect due to some substructure within those hydrosystems in relation to our sampling scheme in two or three sampling sites.

Overall, these results show a strong influence of genetic drift in probably small populations, which might reduce the effectiveness of natural selection (Ferchaud et al. 2020).

The significant difference in allelic richness found among the three islands reflects significant variations in genetic composition across the archipelago, with higher allelic richness in Saint‐Pierre, followed by Miquelon. This result might be due to historical stocking activities in the archipelago, which were conducted mainly in these two islands; in Miquelon, stocking was carried out until 1997 using broodstock originating from *Terre‐Grasse* (*Mirande*), while on Saint‐Pierre, fry production lasted at least until 2012, using broodstock from *Savoyard* and *Goéland* ponds (Briand et al. 2021; Viana et al. 2022). Several studies carried out on brook charr populations in the southern Appalachians or in North Carolina have documented a higher genetic variation in stocked populations following the introduction of individuals from other spatially close wild populations (Létourneau et al. 2018; Gossieaux et al. 2019; White et al. 2023; Smith et al. 2024). In comparison, the Langlade populations were probably much less affected by these actions, which could explain their lower allelic richness. In addition, most of the hydrosystems in Langlade are unable to accommodate anadromous brook charr upstream.

### 4.2 Significant Genetic Structure Among Islands

The most striking result of this study is the occurrence of genetic structure at each spatial scale evaluated, including among the three islands. Each island is separated from the others by the marine environment: Saint‐Pierre and Langlade are separated by an inlet 6 km wide and over 50 m deep, Miquelon and Langlade are linked only by a sandy isthmus, with no freshwater habitat, and the distance between Saint‐Pierre and Miquelon is more than twice that between Saint‐Pierre and Langlade (Cerema and DTAM de Saint‐Pierre et Miquelon 2018). A significant genetic differentiation was therefore expected, as the species has a limited dispersal ability at sea (Castonguay, FitzGerald, and Côté 1982; O'Connell 1982; Castric and Bernatchez 2003). For example, Curry, van de Sande, and Whoriskey (2006) studied the seasonal movements of anadromous brook charr populations in the Laval River (Quebec) for 1 year using acoustic telemetry. They showed that the movements of individuals were restricted to coastal areas (< 500 m from shore), in shallow areas (< 1.7 m), and that the species high co‐tolerance to temperature and salinity was involved in the dispersal capacity and current distribution of populations (Curry, van de Sande, and Whoriskey 2006).

These limited dispersal capacities in the marine environment suggest that contemporary gene flow could mainly be the result of recent anthropogenic actions (i.e., stocking) rather than natural dispersal.

### 4.3 Variable Levels of Connectivity Among Hydrosystems

At the hydrosystem level, a significant genetic structure was observed, with *F*_ST_ similar to those of other brook charr populations in the east of their range (Angers and Bernatchez 1998, *F*_ST_ = 0.37; Hébert et al. 2000, *F*_ST_ = 0.19; Castric, Bonney, and Bernatchez 2001, *F*_ST_ = 0.22; Castric and Bernatchez 2003, *F*_ST_ = 0.11; Pilgrim et al. 2012, *F*_ST_ = 0.14). This moderate structure indicates restricted gene flow, and it would be interesting to assess the impact of environmental and geographical parameters on the genetic differentiation of these populations.

A significant proportion of the overall genetic variation can be attributed to differences among hydrosystems (AMOVA results). As each of these hydrosystems is integrated into a distinct watershed, this result probably reflects the history of the species postglacial recolonization of the archipelago from the Acadian refugium. The importance of watersheds in the differentiation and genetic structuring of brook charr populations has already been highlighted in previous studies (Perkins, Krueger, and May 1993; Danzmann et al. 1998; Castric, Bonney, and Bernatchez 2001; Kazyak et al. 2018). Bruce et al. (2019), in particular, examined the spatial genetic structure of brook charr collected from 18 distinct watersheds in the Northeastern United States using 13 microsatellite loci. As here, they showed a strong subdivision of populations across different watersheds, with a significant proportion of overall genetic variation attributable to watershed boundaries or major drainage basins, reflecting the postglacial recolonization phase of these populations via oceanic routes (Bruce et al. 2019).

Of the different clusters identified with STRUCTURE, two are located in Langlade (i.e., *Anse à Ross*, *Voiles Blanches* and *Maquine* hydrosystems) and are not admixed with other clusters, which indicates that the dispersal from other hydrosystems is very low or nonexistent. In fact, these three hydrosystems are linked to the ocean by impassable waterfalls, preventing the natural return of fish to reproduce. Several studies have shown that physical barriers limit gene flow of brook charr (Poissant, Knight, and Ferguson 2005; Torterotot et al. 2014; Kelson et al. 2015; Timm et al. 2016; Nathan, Kanno, and Vokoun 2017), affecting the genetic connectivity of populations residing in different river systems. For instance, Gomez‐Uchida, Knight, and Ruzzante (2009) by studying the effects of landscape factors on neutral divergence and gene flow in brook charr in *Gros Morne* National Park (Newfoundland, Canada) have demonstrated the influence of waterfalls on genetic diversity and divergence between populations above and below them.

The clusters of Miquelon and Saint‐Pierre are highly admixed which suggests an impact of stocking. In particular, the *Mirande* hydrosystem in Miquelon is strongly admixed with the main cluster of Saint‐Pierre, which makes sense given that the majority of strains used for restocking come from Saint‐Pierre, and *Mirande* has been the subject of several restocking actions using these strains (Briand et al. 2021; Viana et al. 2022).

### 4.4 Genetic Differences Within Hydrosystems

Significant genetic differences were found within three of the archipelago hydrosystems: *Cap au Diable*, *Thélot* (Saint‐Pierre) and *Sylvain* (Miquelon). At *Cap au Diable* and *Thélot*, genetic differentiation was found between freshwater habitats. At *Cap au Diable*, this includes a marsh (median portion) and a downstream river that flows into the ocean via an impassable waterfall. At *Thélot*, genetic differentiation was found between the upstream river and the median pond. In *Sylvain*, genetic differentiation was observed between brook charr located in a pond upstream and in the median part of the hydrosystem, as well as between this upstream pond and the most downstream site.

The brook charr is one of the most genetically structured animal species (Ward, Woodwark, and Skibinski 1994). Observations of very fine‐scale structuring have already been demonstrated in other native populations of the species (Angers and Bernatchez 1998; Angers et al. 1999). Hébert et al. (2000) studied the influence of hydrographic structure on the genetic organization of 24 brook charr populations from three Canadian national parks (Kouchibouguac, Fundy and Forillon). They showed that each of them mated nonrandomly, even when they were in the same watershed and sometimes only a few kilometers apart, less than 5 km (Hébert et al. 2000). These results, and those of our study, therefore, show that native populations of freshwater resident brook charr can develop distinct reproductive units even at a small spatial scale, which may have strong implications for their management (e.g., regulation protecting the genetic integrity of reproduction units).

## 5 Conclusion

Our results highlight a complex pattern of genetic structure among brook charr populations in a tiny archipelago. The lack of a clear pattern of isolation by distance, combined with predominantly interisland gene flow and complete genetic isolation of some hydrosystems, suggest that this contemporary genetic structure is primarily the result of natural demographic processes during the species postglacial colonization altered by recent stocking actions.

It is crucial to integrate these results into future management decisions. The populations showing both isolation and low levels of diversity, as well as their associated habitats, should be protected in priority and restocking should be restricted as much as possible to preserve the local adaptive potential of this very important angling species in Saint‐Pierre and Miquelon.

## Ethics Statement

All captures were conducted in compliance with legal authorizations granted by the relevant authorities, including the Canadian Council on Animal Care (UQAR CPA‐89‐22‐246) and to the *Direction des Territoires, de l'Alimentation et de la Mer* (prefectural permit no. 349).

## Conflicts of Interest

The authors declare no conflicts of interest.

## Supporting information


Data S1.

## Data Availability

Data for this study are available at doi: 10.5061/dryad.vdncjsz4r.
