# Supplementary material for: Fine‐Scale Genetic Structure of Small Fish Populations in Islands: The Case of Brook Charr Salvelinus fontinalis (Mitchill, 1814) in Saint‐Pierre and Miquelon (France)
Source: Evol Appl. 2025 Jan 16;18(1):e70041. doi: 10.1111/eva.70041 (PMC11736641; doi:10.1111/eva.70041)

Table S1. Primer sequences, primer-specific annealing temperature (T*a*) in °C, fragment size range (*bp*), and fluorochrome added for the twelve microsatellite *loci* used in this study. The four multiplexes are distinguished by bold lines.

Table S2. Linkage disequilibrium tests in each hydrosystem using the log-likelihood ratio test implemented in the GENEPOP v4.0 program. Only pairs of *loci* with a significant *p-value* (< 0.05) are indicated.

| **Hydrosystem** | ***Locus* 1** | ***Locus* 2** | ***p-value*** |
| --- | --- | --- | --- |
| *Mirande* | B52 | C113 | 0.017 |
| *Carcasse de l'ouest* | C115 | Sfo308 | 0.009 |
| *Carcasse de l'ouest* | D75 | C129 | 0.015 |
| *Bellevue* | C115 | C86 | 0.034 |
| *Bellevue* | Sfo308 | B52 | 0.025 |
| *Bellevue* | C115 | D91 | 0.032 |
| *Sylvain* | Sfo308 | C86 | 0.017 |
| *Sylvain* | D75 | D100 | 0.042 |
| *Sylvain* | C86 | D100 | 0.001 |
| *Sylvain* | C115 | D91 | 0.036 |
| *Cap au diable* | C115 | C38 | 0.038 |
| *Cap au diable* | D75 | C38 | 0.029 |
| *Thélot* | C115 | D100 | 0.010 |
| *Thélot* | C86 | D100 | 0.018 |
| *Savoyard* | Sfo308 | C86 | 0.016 |
| *Savoyard* | C38 | B52 | 0.031 |
| *Savoyard* | B52 | D91 | 0.049 |
| *Savoyard* | D75 | C129 | 0.014 |
| *Savoyard* | D100 | C113 | 0.030 |
| *Voiles Blanches* | B52 | D100 | 0.051 |
| *Anse à Ross* | C115 | C113 | 0.020 |
| *Debons* | C115 | D75 | 0.045 |
| *Debons* | Sfo308 | C38 | 0.049 |
| *Debons* | D75 | C86 | 0.013 |
| *Debons* | C38 | C86 | 0.006 |
| *Debons* | C38 | D91 | 0.042 |
| *Debons* | C86 | D91 | 0.042 |
| *Debons* | C38 | C129 | 0.025 |
| *Debons* | C86 | C129 | 0.004 |
| *Debons* | B52 | C129 | 0.041 |
| *Debons* | D100 | C129 | 0.014 |
| *Debons* | C86 | C113 | 0.013 |
| *Debons* | C129 | C113 | 0.018 |

Fig. S1. Results of the analysis of the Delta *K* statistics. *K* = 4 appears to be the uppermost hierarchical level of genetic structure.

*
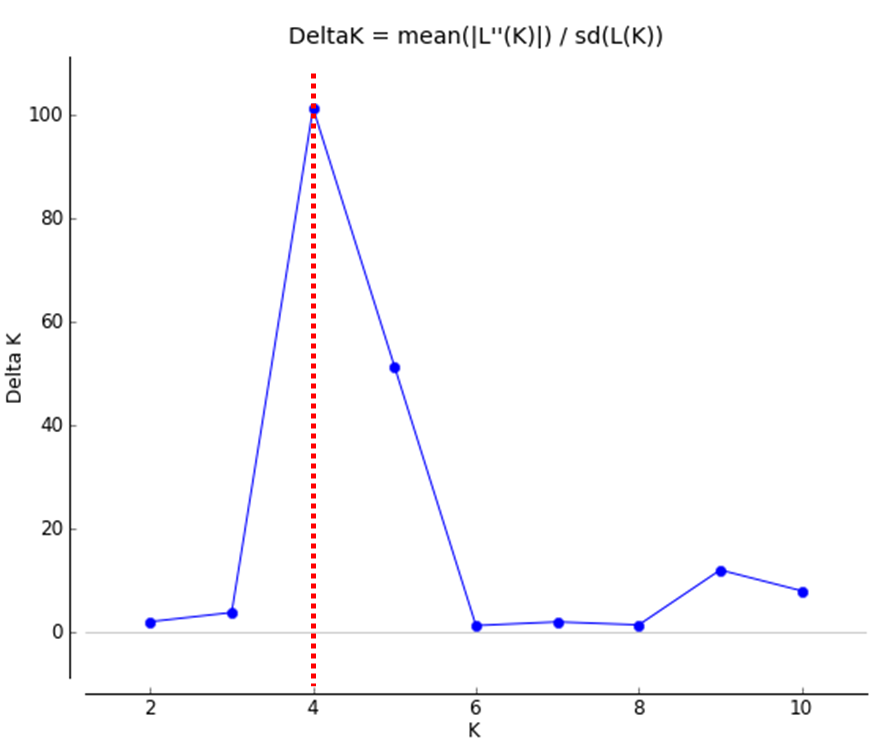
*

Fig. S2. Pairwise genetic distances (*F*_ST_/1- *F*_ST_) as a function of logarithmic coastal distances between hydrosystems (km).


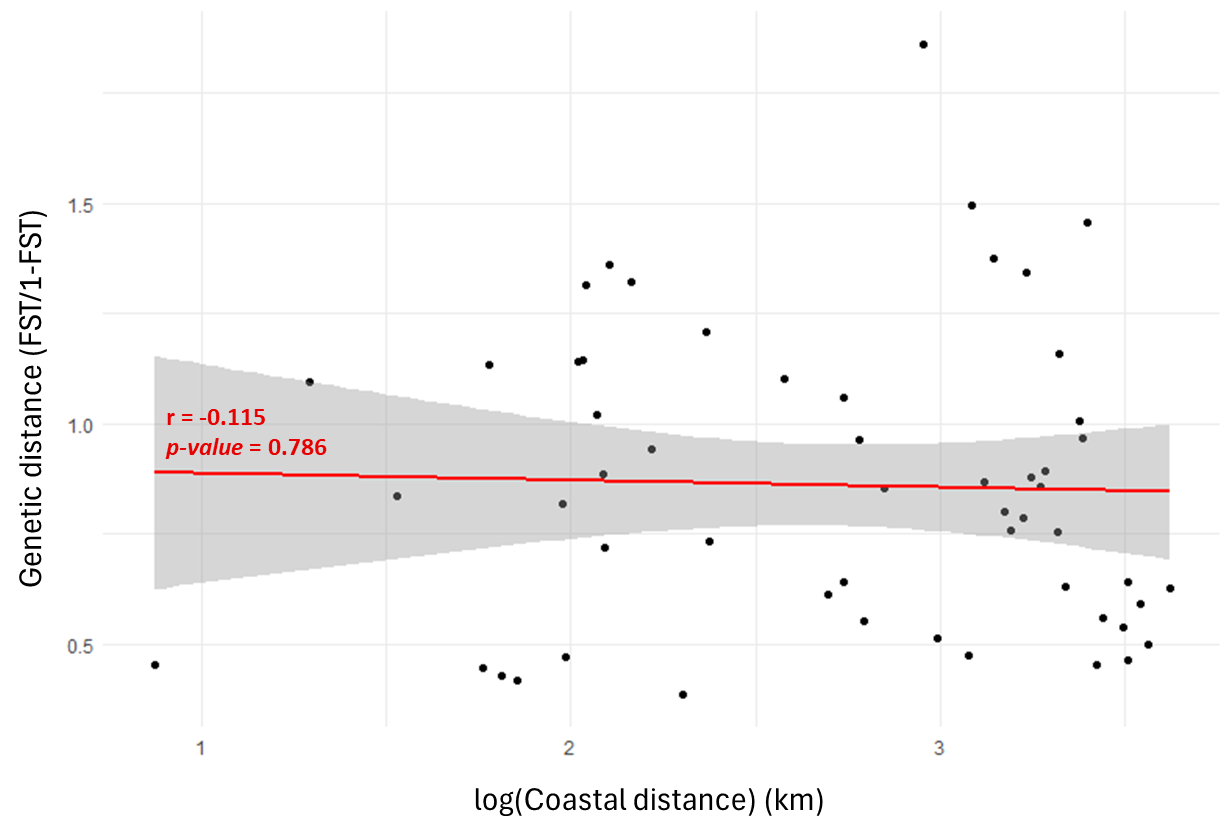

Supplement: Supplementary file 1 — Data S1. [file EVA-18-e70041-s001.docx]
